# Tumor-like herpes simplex infection in an HIV patient^☆^^☆☆^

**DOI:** 10.1016/j.abd.2020.12.005

**Published:** 2021-05-15

**Authors:** Lula María Nieto-Benito, Ángel Manuel Rosell-Díaz, Ana Pulido-Pérez, Ricardo Maria Suárez-Fernández

**Affiliations:** Department of Dermatology, Hospital General Universitario Gregorio Marañón, Universidad Complutense de Madrid, Madrid, Spain

**Keywords:** Herpes genitalis, Herpes simplex, HIV infections, Infections, Skin diseases

## Abstract

A 56-year-old male, HIV-positive, presented with a 3-day history of multiple indurated erythematous nodules with superficial and well-defined erosions on his right gluteus. Skin biopsy showed ballooning-necrotic keratinocytes and cultures were positive for herpes simplex 2. Genital herpes simplex infection recurrences may not be restricted to the anterior part of the genitalia and clinical presentation in the lumbar area or gluteus must be differentiated from varicella-zoster virus infection. Tumor-like presentation is a very rare manifestation of HSV cutaneous infection. It is important to take this morphological variant into consideration not to delay the diagnosis of a viral infection, especially in an immunosuppressed patient.

A 56-year-old male, HIV-positive, presented with a 3-day history of painful lesions on his right gluteus. He denied a prior diagnosis of genital herpes. The patient had been on antiviral medication since diagnosis and his viral load was undetectable with a CD4 cell count of 942 cells/µL. He denied any systemic symptoms. During the physical examination, multiple indurated erythematous nodules with superficial and well-defined erosions were noted (Fig. 1). Skin biopsy was performed for histopathological studies and microbiological cultures. Blood analysis was within normal values. Biopsy of one of the lesions showed ballooning-necrotic keratinocytes and acantholysis with inflammation extending to subcutaneous tissue (Fig. 2). A diagnosis of nodular cutaneous and herpes simplex infection was made. Cultures supported the diagnosis as they were positive for Herpes simplex 2 and negative for bacteria mycobacteria and fungi. Valacyclovir was initiated and followed by complete resolution within 7-days.

**Figure 1:**
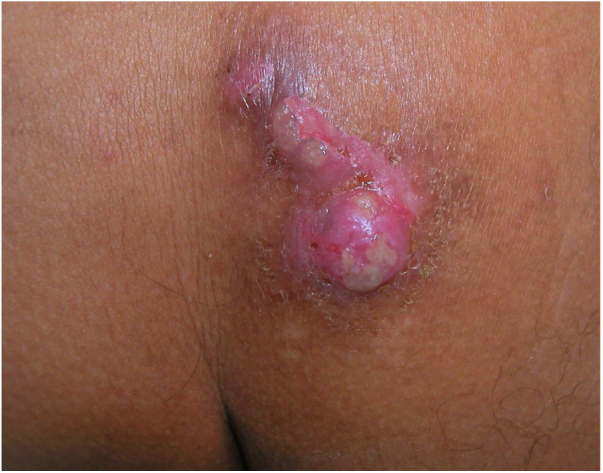
Multiple indurated, erythematous nodules with superficial erosions with irregular, geographic borders.

**Figure 2:**
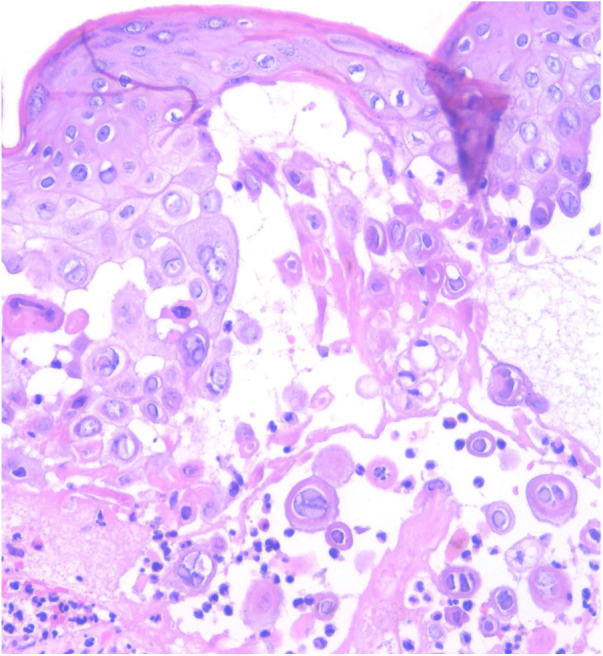
Keratinocytes with ballooned nuclei, compacted, marginalized chromatin into the nuclear periphery, with a ground-glass appearance and giant multinucleated keratinocytes (Hematoxylin & eosin, ×100).

Genital herpes simplex infection recurrences may not be limited to the anterior part of the genitalia and clinical presentation in the lumbar area or gluteus must be differentiated from a varicella-zoster virus infection.^1^ HSV polymerase chain reaction-based testing and cell culture of the lesion can be helpful to determine the causative virus.^1^, ^2^ Tumor-like presentation is a very rare manifestation of HSV cutaneous infection.^3^ It is important to take this morphological variant into consideration not to delay the diagnosis of a viral infection, especially in an immunosuppressed patient.^4^ Nodular genital herpes simplex is a very rare form of clinical presentation; only one case has been reported in the literature.^3^, ^5^ The authors describe a case of cutaneous HSV (herpes simplex virus) infection in an HIV patient that clinically presented with a nodular configuration, mimicking a tumor, supported by histopathology and microbiological cultures.

## Financial support

None declared.

## Authors' contributions

Lula María Nieto-Benito: Have conceived and designed the analysis; collected the data; contributed data or analysis tools; performed the analysis; written the paper and approved its final version.

Ángel Manuel Rosell-Díaz: Have collected the data and approved its final version.

Ana Pulido-Pérez : Have conceived and designed the analysis; collected the data; contributed data or analysis tools; performed the analysis; written the paper and approved its final version.

Ricardo Maria Suárez-Fernández : Have conceived and designed the analysis; collected the data; contributed data or analysis tools; performed the analysis; written the paper and approved its final version.

## Conflicts of interest

None declared.
